# Wavelength‐Dependent 3D Printing: Introducing 3D Printed Action Plots

**DOI:** 10.1002/adma.202523664

**Published:** 2026-04-16

**Authors:** Federica Sbordone, Lauren Geurds, Joshua A. Carroll, Yanan Xu, Alicia K. Finch, Filip Petko, Andrzej Świeży, Joanna Ortyl, Christopher Barner‐Kowollik

**Affiliations:** ^1^ Institute of Functional Interfaces (IFG) Karlsruhe Institute of Technology (KIT) Hermann‐von‐Helmholtz‐Platz 1 Eggenstein‐Leopoldshafen Germany; ^2^ School of Chemistry and Physics Centre for Materials Science Queensland University of Technology (QUT) 2 George Street Brisbane Queensland Australia; ^3^ Central Analytical Research Facility Queensland University of Technology 2 George Street Brisbane Queensland Australia; ^4^ Department of Biotechnology and Physical Chemistry Faculty of Chemical Engineering and Technology Cracow University of Technology Warszawska 24 Cracow Poland

**Keywords:** material properties of 3D printed objects, photochemical action plots, wavelength resolved 3D printing

## Abstract

Light‐driven additive manufacturing is a key technology that relies on the ability of photons to effectively initiate the curing of a photoresin. We have previously demonstrated via photochemical action plots that irradiating a chromophore at its maximum wavelength of absorption does not necessarily lead to a photochemical process with the highest efficiency. Today, action plots are considered a powerful methodology to access wavelength‐resolved photochemical reactivity. However, there is currently no study that translates the knowledge gained from solution‐based photopolymerization action plots to light‐driven 3D printing. Herein, we investigate the reactivity of a photoresin system and the formation of a cured polymer network using a stereolithography 3D printing set‐up coupled with a monochromatic wavelength tunable laser system, carefully determining the effect of disparate monochromatic wavelengths on the printing outcome in analogy to solution photochemical action plots. We find that although the reactivity of the resin used herein, and subsequent material properties, are not fully aligned with the solution action plot of the solution photopolymerization using the same photoinitiator, a 3D Printed Action Plot (3D‐PAP) revealed that curing the photoresin at longer wavelengths results in efficient 3D printing and superior mechanical properties in line with the red‐shifted highest reactivity observed for the solution photopolymerization.

## Graphical abstract

Photochemical action plots – wavelength‐dependent reactivity studies of photoactive molecules – can show a significant mismatch between the peak of photochemical action and maximum absorbance in photopolymerization systems in solution. How this knowledge translates to photocuring in light‐driven 3D printing remains unexplored. Herein, the wavelength‐dependent photochemical crosslinking of a photoresin is investigated, pioneering the 3D Printed Action Plot (3D‐PAP) methodology.

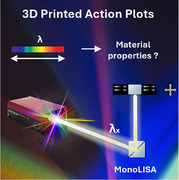

## 1 Introduction

The field of light‐driven 3D printing has made formidable strides in the last decade [1, 2, 3], not only with regard to the printers that have been developed – including the most recent developments of light‐sheet and volumetric printing [4, 5]– but also in the types of chemistries that constitute the resins [6, 7]. Most recently, efforts have concentrated on how to achieve such complex structures from one photoresin [8, 9, 10, 11, 12, 13, 14], guided by the need to develop light‐driven 3D printing methodologies that are capable of producing multi‐material objects in a single fabrication step. Perhaps the most well‐known and established process for achieving material variety within one 3D fabricated design is grey‐tone lithography [15], where a variation in the photon flux leads to different degrees of curing within the resin and thus regions of different hardness within the 3D printed object [16, 17]. While the field of grey‐tone lithography is still developing further, recent progress has focused on employing multiple wavelengths to control the curing of a resin that contains chemistries that can be – ideally orthogonally – activated with disparate colors of light [18, 19, 20, 21, 22, 23]. In both cases, selecting the optimum wavelengths and light intensity is critical to enable efficient 3D printing.

Over the past decade, we have demonstrated that the absorption spectrum of a chromophore holds only limited information for predicting which wavelength will progress a specific reaction trajectory most efficiently [24, 25, 26, 27]. To obtain information on the wavelength‐dependent conversion or – ideally – the quantum yield of a reaction, a so‐called photochemical action plot must be recorded [28, 29], which probes the efficiency of the reaction wavelength‐by‐wavelength using a monochromatic tunable laser. An identical number of photons is delivered for each specific monochromatic wavelength, and the chemical reactivity is subsequently mapped via chemical characterization techniques such as NMR or UV/Vis spectroscopy. Since the initial development, a wide range of photochemical systems has been mapped, many showing a significant mismatch between the peak of photochemically induced action of the system and the maximum absorbance [30, 31, 32, 33].

While it was initially unclear why many photochemical reactions show a strong mismatch between absorptivity and reactivity [28], a mechanistic picture is currently emerging that centers on the microenvironment surrounding each individual chromophore in solution, leading to a variation in the energetic distance between the ground and first excited state at the red‐edge of the absorption spectrum [29]. Critically, this reduction in energy gap can be accompanied by enhanced excited‐state lifetimes that can lead excited chromophores to react with a higher probability through increased crossing into the triplet state. Thus, the wavelength‐dependent quantum yields for a specific reaction are strongly dependent on its surrounding environment.

Remarkably, recording an action plot of each photoactive component of the desired resin in solution has enabled the design of systems that are orthogonal [20], as well as synergistic [34], cooperative [18] and antagonistic [35], and the fabrication of multi‐materials starting from only one, albeit complex, photoresin. For example, we have recently introduced the tunable laser set‐up MonoLISA that allows printing of light‐degradable and light‐stable geometries from one photoresin simply by changing the color of light of the incident laser beam [20]. Here, the two chemistries can be activated fully wavelength‐orthogonally, meaning that the order in which they are applied can be freely selected, with the result that only one component of the resin is cured. Other examples, including those from the Boydston group, have demonstrated that the different material properties can also be obtained in situations where one resin is cured by the less energetic color, while both resin components are activated with the more energetic color of light [36].

Even though action plots of photoactive molecules in solution have been pivotal for the design of such photoresins, the curing of a photoresin during 3D printing occurs in a drastically more complex (micro)environment. While the typical action plot set‐up allows for control of reaction conditions (e.g. presence of oxygen, choice of solvent and concentrations, negligible scattering), critical factors such as the interaction between chemical components of a photoresin, concentration requirements, presence of oxygen, light penetration depth and scattering are variables that could significantly influence the behavior of chromophores when transitioning from systems in solution to 3D printing systems.

Herein, we present a wavelength‐resolved analytical method for the evaluation of the curing performance of a photoresin akin to a solution‐based action plot, which we term ‘3D Printed Action Plot’ (3D‐PAP). As the principle of the action plot method relies on the irradiation of the system of interest with an identical number of photons at all investigated wavelengths, any difference in reactivity – here network formation – and subsequent difference in properties of the resulting structures will be entirely dependent on the wavelength. In our approach, the difference in network formation will be correlated with the change in wavelength, rather than variations of light intensity, as exploited in grayscale lithography. We envisage that the thorough investigation of the wavelength‐dependent curing will not only widen the window of optimum printing parameters of photoresins, but also provide an exciting opportunity to expand the toolbox of material properties engineering.

Critically, since many organic photoresponsive molecules absorb light in the UV region, or typically below 400 nm [37], the relatively high energy irradiation required limits its application (i.e., in biological fields) and makes it challenging to move away from exploiting photochemistry as a surface phenomenon, due to the limited penetration depths of short wavelengths. 3D‐PAPs hold the potential of broadening the scope of 3D printed structure applications, especially considering that action plots of photopolymerizations usually reveal significantly red‐shifted peak reactivities, as evident in earlier studies [26, 38]. Therefore, the prospect of optimizing the efficiency of the desired 3D printing process holds the advantage of using much milder wavelengths and, thus, less energy, and gaining penetration depth.

We commence our exploration of achieving wavelength‐dependent material properties in printed objects by establishing a photochemical action plot for a radical photoinitiator. We subsequently use the same photoinitiator in our resin formulation to assess the printability and the resulting material properties of (3D) printed objects over a range of wavelengths emitted from a tunable laser in an SLA printer system.

## 2 Results and Discussion

Photoinitiators play a key role in light‐initiated radical polymerization. They should efficiently start the polymerization process – by virtue of the generated primary radicals – and be highly sensitive to the desired light source. Most commercially used photoinitiators are active below 400 nm, which represents a significant limitation for applications that require the use of non‐harmful wavelengths or higher resin penetration depths. Over the last few years, efforts have been focused on the development of new red‐shifted free radical initiating systems that are active upon longer (safer) wavelength irradiation. The Ortyl group recently developed the red‐shifted photoinitiator (*E)*‐1,1‐dimethoxy‐4‐(4‐methylsulfanylphenyl)‐1‐phenyl‐but‐3‐en‐2‐one [DMPP‐(L)‐SMe] [39], based on the well‐known 2,2‐dimethoxy‐2‐phenylacetophenone (DMPA) structural motif.

**FIGURE 1:**
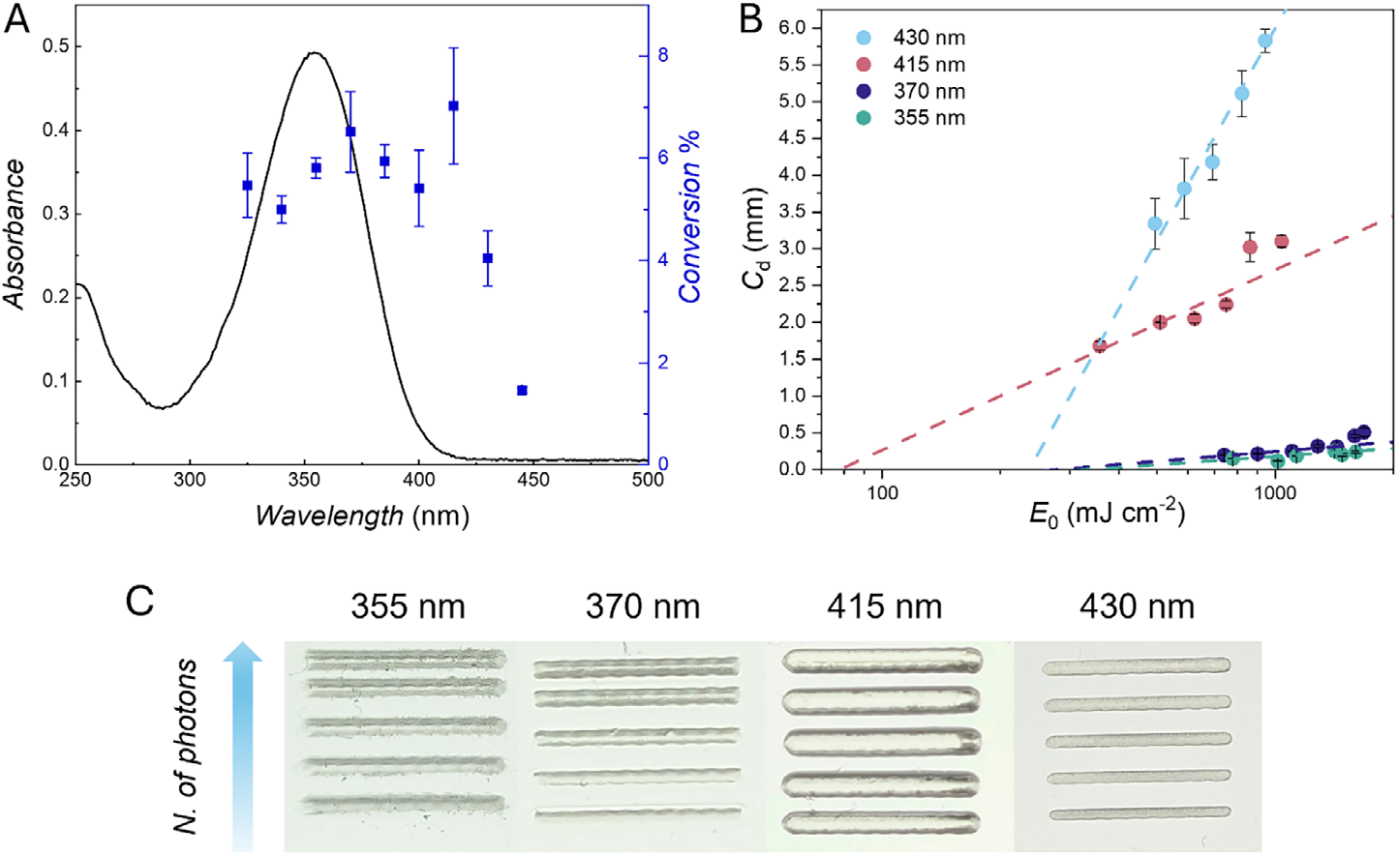
(A) Photopolymerization action plot employing the photoinitiator DMPP‐(L)‐SMe in bulk methyl methacrylate (MMA) at ambient temperature. The photoinitiator concentration was close to 5∙10^−3^ mol L^−1^ in each case, and 4.56∙10^19^ photons were delivered at each monochromatic wavelength. The entire data set is available in the Section S2. (B) Jacobs working curves of the photoresin. The photoresin composition is 0.5 wt.% of the DMPP‐(L)‐SMe photoinitiator and 25 wt.% pentaerythritol triacrylate (PETA) in poly(ethylene glycol) diacrylate *M*_n_ = 700 g mol^−1^ (PEGDA 700). Refer to Supporting Information Section S4 for experimental details and equations. Experiments in panels A and B are performed in triplicate; the error bars represent standard deviation. (C) Preliminary Printed Action Plot for optical inspection at the four wavelengths of interest. The number of photons is constant across corresponding lines and decreases by 10% (details in Supporting Information Section S5) from top (1.84·10^16^ photons s^−1^) to bottom (1.02·10^16^ photons s^−1^).

Initially, we recorded a photopolymerization action plot of DMPP‐(L)‐SMe and methyl methacrylate (MMA) in solution (Figure 1A). The action plot analysis reveals that the maximum reactivity wavelength of DMPP‐(L)‐SMe does not correspond with the maximum absorbance at 355 nm, and a pronounced red‐shifted reactivity up to 430 nm is observed, with a region of comparable or higher reactivity found at around 415 nm.

To investigate whether these findings can be translated to the printing of cured polymer networks, we initially designed preliminary twin wavelength‐resolved experiments in a printed format, constituting the essence of printed action plots. A photoresin constituted of DMPP‐(L)‐SMe (0.5 wt.%), pentaerythritol triacrylate (PETA) (25 wt.%) in poly(ethylene glycol) diacrylate *M*_n_ = 700 g mol^−1^ (PEGDA 700) was prepared and used for printing lines at four wavelengths, selected according to the following criteria: 355 nm as the absorption maximum of the photoinitiator, 370 nm as it shows a similar conversion to the λ_max_ according to the solution action plot, albeit at a red shifted wavelength, 415 nm as it displays comparable or higher reactivity at negligible absorptivity and – finally – 430 nm as the most red‐shifted wavelength with appreciable reactivity yet lower conversion. The photoresin was poured into a mold to form a layer of 1 mm in height, secured onto a glass slide as a printing platform for the MonoLISA printer (set up details in Supporting Information Section S3). A set of five lines per wavelength was printed – as an initial preliminary experiment –progressively decreasing the number of photons delivered each time by close to 10%, with the top line receiving the highest number of photons and the bottom line receiving the lowest number of photons in each set (Figure 1C). Importantly, the number of photons delivered across the corresponding lines of the different sets was kept constant, so that any observed difference between prints is entirely wavelength dependent. A stark difference is immediately noticeable between the sets of lines cured at 415 and 430 nm, and the shorter wavelengths 370 and 355 nm (Figure 1C). In terms of size and printing definition, the lines printed at 355 and 370 nm show some irregularities and appear slightly opaque, probably due to scattering effects. A thickness of close to 1.3 mm and height ranging from 0.2 to 0.26 mm was measured for the lines printed with 1.42·10^16^ photons s^−1^ (third line in Figure 1C, SEM images in Figure S10). In contrast, the lines printed at 415 and 430 nm appear well‐defined and clear, hinting at a much‐reduced scattering effect and, therefore, at likely different network properties. The lines printed with 1.42·10^16^ photons s^−1^ show a thickness of approximately 0.95 mm and increased height values (close to 1 mm), non‐surprisingly due to increased penetration depth at longer wavelengths. It is important to note that the height of the photoresin layer in the mold in the setup used for this experiment is 1 mm, limiting the maximum line height possible. In addition, decreasing the number of photons delivered to the 415 nm set seems to have negligible effects on the printing of the lines in the investigated range, whilst the same decrease at 355, 370, and 430 nm results in progressively thinner lines.

Encouraged by these preliminary results, further investigation of the wavelength's influence on printing behavior was performed by measuring Jacob working curves of the same photoresin cured at different wavelengths (Figure 1B). The working curve relates the radiant exposure at the surface of the photoresin to the thickness of the cured polymer as follows:

$$C_{d}=D_{p}\ln\left(\frac{E_{0}}{E_{c}}\right)$$

where *C*_d_ is the curing depth (i.e., thickness) of the polymer, *D*_p_ is the optical penetration depth of the light source into the resin, *E*_0_ is the incident radiant exposure, and *E*_c_ is the critical energy or minimal radiant exposure necessary to transform (i.e., cure) the liquid resin into a solid. A semi‐logarithmic plot of *C*_d_ *vs. E*_0_ produces a linear curve with a slope of *D*_p_ and a x‐intercept of *E*_c_ [40].

Squares of 2 × 2 mm were printed at increasing *E*_0_ by increasing the laser power (the photoresin layer depth in the vat was close to 2 cm). It is evident for all curves that the data do not perfectly fit a single logarithmic regression line (R^2^ < 98), with non‐linear behavior observed at higher exposures. These deviations are usually due to a change in the optical properties of the resin as it cures. In this specific case, the refractive index of photocured PEGDA is larger than that of non‐cured PEGDA, causing optical self‐focusing and redirecting photons from the outer portions of a Gaussian‐shaped focused laser beam to its centerline [41]. The increased photon dose cures more resin, increasing *C*_d_. While considering that the accuracy of the exact values for *D*_p_ and *E*_c_ may be somewhat affected by the deviation from the linear behavior, we clearly observe an increase in *D*_p_ as a function of wavelength, as expected when using longer wavelengths. Notably, 415 nm allows curing of the photoresin with significantly lower radiant exposure (*E*_c_ = 119 mJ cm^−2^, more than 4 times lower than the λ_max_), followed by 430, 370 and 355 nm which move toward higher critical energy values (*E*_c_ of 214, 344, and 485 mJ cm^−2^ respectively), in accordance with what observed in the set of lines printed at 355, 370 and 430 nm that show progressive thinning at lower printing powers in contrast to the set printed at 415 nm. Taken together with the results shown in the polymerization action plot (Figure 1A), the lowest critical energy observed at 415 nm suggests higher reactivity of the initiator at this wavelength.

### 2.1 2D Printed Action Plot

**FIGURE 2:**
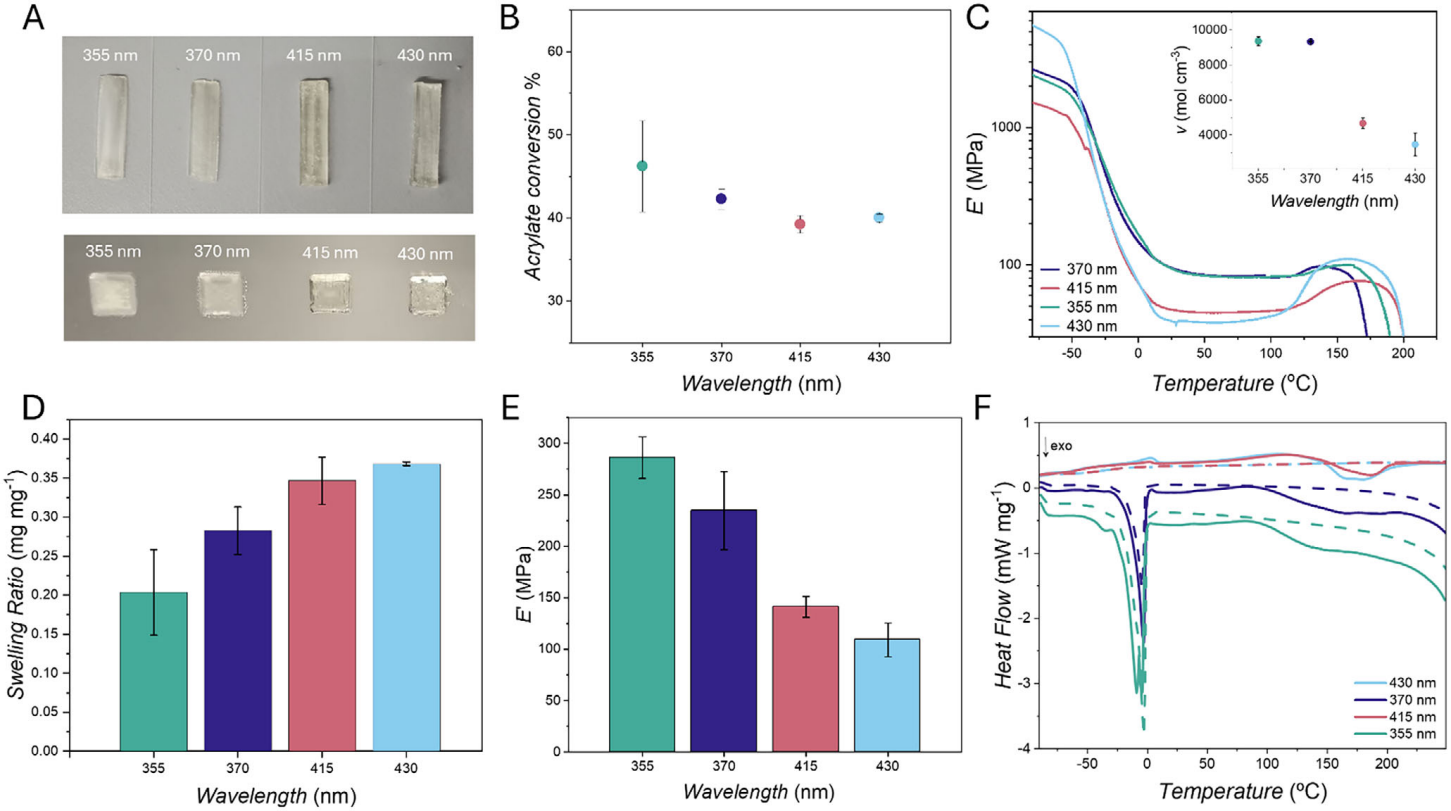
Characterization of one‐layer prints. (A) Examples of 2D Printed Action Plots, 5 × 20 mm rectangles (top) and 5 × 5 mm squares (bottom). All structures were printed with the same photoresin and are exposed to 1.42·10^16^ photons s^−1^. Printing conditions are reported in Supporting Information Section S3 alongside the G‐Code used for each 2D Printed Action Plot in Figure S9. Characterization of the cured structures via (B) FTIR, (C) DMA, (D) Swelling in water, and (E) Nanoindentation was performed in triplicate. The error bars represent standard deviation. Representative spectra are reported in panel (C) for DMA analysis; the inset displays the calculated crosslinking density (*v*) in the rubbery regime at 50°C. (F) DSC representative thermograms recorded from −90 to 250°C, first and second heating runs are displayed (solid and dashed lines respectively).

Comparison of the data obtained after photocuring the resin delivering the same number of photons (Figure 1C) – or the same incident radiant exposure (Figure 1B) – leads to the hypothesis that printing at 415 nm might be a more optimum choice in terms of lower energy requirements and comparable or even potentially higher photoinitiator reactivity according to the action plot (Figure 1A). The action plot suggests comparable or higher conversion – and therefore higher crosslinking efficiency and tougher materials – at 370 and 415 nm, with comparable or slightly lower reactivity at 355 nm, and yet lower reactivity at 430 nm. Notably, the printed lines appear markedly different not only in their size, which is related to beam size, scattering, and penetration depth. The prints at 355 and 370 nm appear opaque, hinting at the presence of crystallites that scatter light [42] in contrast to the clearer structures obtained at 415 and 430 nm (Figure 2A), suggesting different network and materials properties. Further characterization of the cured structures is thus necessary to test our hypothesis and establish whether the difference in reactivity observed in the solution action plot translates to network formation in printing conditions, and if the material properties of the cured structures are related to wavelength‐dependent reaction efficiency.

To deeper characterize their network and material properties, 2D Printed Action Plots constituted by one‐layer structures printed using the same photoresin, the same G‐Codes and delivering the same number of photons at all wavelengths (examples in Figure 2A) were carried out, and the printed structures analyzed via Fourier Transform Infrared spectroscopy (FTIR), Dynamic Mechanical Analysis (DMA), Differential Scanning Calorimetry (DSC), Nanoindentation and swelling tests.

Surprisingly, FTIR analysis (Figure 2B) shows conversions of the acrylates' double bond (C═C peak = 810 cm^−1^, C═O reference peak = 1720 cm^−1^) to be comparable for all wavelengths (representative spectra are provided in Figure S11). Values appear within error for 355 and 370 nm, with average conversions of 46% and 42% respectively, and the average conversion of the 415 and 430 nm prints only slightly lower (39% and 40% respectively). Possible explanations for the double bond conversions obtained include (*i*) during curing conversion has reached the plateau regime and converged at all wavelengths, whilst in the polymerization action plot in solution conversion is intentionally kept very low to stay in the linear conversion regime, and (*ii*) that lower double bond conversion (in particular at 415 nm) can be attributed to higher initiation efficiency in the system investigated, as it is a curable system [43]. In the case of photocrosslinking processes, efficient initiation initially accelerates polymerization, which then limits the diffusion of monomers/macroradicals as it progresses due to the restricted mobility of the crosslinked polymer chains formed. Thus, reaction diffusion becomes the dominating termination mechanism already at relatively low conversions [43].

Swelling tests in water, however, reveal that the swelling ability of the structures significantly increases when printing at longer wavelengths (Figure 2D). Higher swelling ratios were obtained for the prints cured at 415 and 430 nm with comparable values, whilst the values are lower for the structures cured at 355 and 370 nm. A lower swelling ratio is generally attributed to more densely crosslinked networks. Considering the similar conversions for these networks obtained from the FTIR study, an alternative explanation for the swelling behavior trend could be related to the homogeneity/inhomogeneity of the networks rather than their crosslinking density [44, 45]. Similarly, DMA analysis (Figure 2C) demonstrates that the prints obtained at 355 and 370 nm have comparable elastic moduli in the rubbery regime and, therefore, crosslinking densities (*v*), which were calculated according to the rubber elasticity theory for ideal networks (inset in Figure 2C) [46]:

$$v=\frac{{E^{\prime }}_{R}}{3RT}$$

where *E’*_R_ is the storage modulus in the rubbery plateau region, *R* is the universal gas constant, and *T* is the absolute temperature. The crosslinking density decreases for the structures cured at 415 nm and decreases further for the prints at 430 nm. The different mobility of the crosslinked chains described by the magnitude of the elastic modulus in the rubbery regime may be influenced not only by the number of crosslinking points, but also by their homogenous/inhomogeneous distribution within the network.

Further investigation of the networks via DSC of the structures cured at 355 and 370 nm revealed sharp exothermic peaks around −7°C and −3°C, respectively (Figure 2F), attributed to cold crystallization, which is favored by the presence of longer strands between junctions [47]. Glass transitions were not detectable with DSC for 355 and 370 nm cured samples (DMA analysis in Figure S12 shows glass transitions for all wavelengths). A different profile results from DSC analysis of the prints obtained at 415 and 430 nm. In both DSC curves, a glass transition is visible together with two consecutive endothermic melting peaks, the first at close to 2°C and a broader one centered around 110°C, of significantly lower magnitude. The presence of double melting peaks is attributed to a recrystallization process. Under slow heating conditions, melting of ordered structures (crystals or ordered melt) and their recrystallization proceed simultaneously (first melting peak) [48]. Finally, the recrystallized crystals, which are larger than the original ones, melt at a higher temperature (second melting peak). The recrystallization process of the structures cured at 415 and 430 nm is indicative of the presence of networks that are ordered to an extent, yet with strand mobility not sufficient to allow the formation of crystalline structures comparable to the networks cured at 355 and 370 nm.

Finally, the mechanical properties of the 2D prints were characterized via nanoindentation (Figure 2E). A progressive decrease in elastic modulus is observed when curing at longer wavelengths, with the networks cured at 355 nm displaying elastic modulus values two times larger than the ones cured at 415 nm and almost three times larger than the networks cured at 430 nm. A higher elastic modulus is generally indicative of more densely crosslinked networks. Taking into account that FTIR analysis shows a difference in average double bond conversion of just 7%–6% between 355 nm and the 415 and 430 nm samples, respectively, the unexpectedly large difference in mechanical properties can be explained by the differences in network topology, such as the presence of crystallites in the structures cured at 355 and 370 nm. As previously observed when comparing heterogeneous acrylate networks with thiol‐ene based homogenous networks [45], the presence of heterogeneously distributed cross‐linking points results in densely crosslinked areas and more loosely crosslinked areas characterized by longer and more mobile polymer chains. Such mobility allows for reorganization of the chains into more ordered structures, including crystallites, increasing the stiffness of the materials. In the case of more homogenous networks, chain mobility is less favored by the presence of more homogeneously distributed crosslinks, preventing the formation of ordered structures.

**FIGURE 3:**
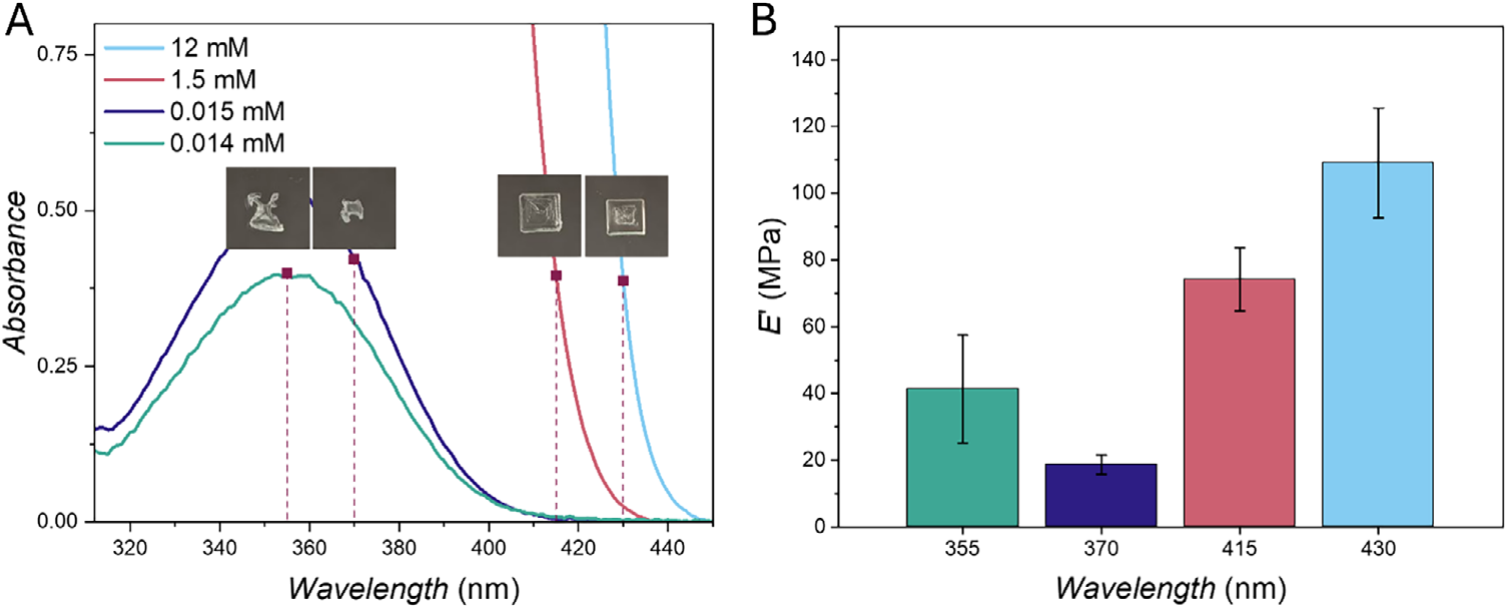
(A) UV–vis spectra of all photoresins with *A* = 0.4 at the wavelength of interest and corresponding printed structures. The photoresins (25 wt.% of PETA in PEGDA 700) with photoinitiator concentrations of 0.014, 0.015, 1.5, and 12 mm were printed at 355, 370, 415, and 430 nm, respectively, delivering 1.42·10^16^ photons s^−1^. (B) Nanoindentation analysis of the 2D Printed Action Plot obtained printing with an equal number of photons absorbed. Measurements are performed in triplicate; error bars represent standard deviation.

The difference in absorbance of the photoresin at the four wavelengths needs to be considered as well for an accurate comparison of wavelength‐dependent printing characteristics. Even though action plots have demonstrated that maximum absorbance is not necessarily correlated with maximum reactivity, it is essential to keep in mind that the absorption event must occur for any photochemical reaction to be possible. What action plots show is that photons have different efficiencies at different wavelengths, as most times the photons absorbed at red‐shifted wavelengths yield a higher number of photochemical events (higher quantum yield) than the same number of photons absorbed at the maximum absorbance wavelength, resulting in a lower number of desired photochemical events (lower quantum yield). However, in the printed action plots described so far, the number of photons delivered is identical, but the number of photons absorbed is different, due to the different absorbance properties of the resin at different wavelengths. To obtain a more accurate comparison of reactivity amongst wavelengths, it is necessary to analyze the efficiency of the photochemical reaction at different wavelengths when the same number of photons is not only delivered, but also absorbed at all wavelengths. Namely, the resin is able to absorb more photons at 355 and 370 nm than at 415 and 430 nm, since the absorbance of the resin is significantly lower at 415 and 430 nm (Figure 3A). Thus, even when delivering the same number of photons at all wavelengths, the number of photons absorbed and potentially initiating photocleavage of the initiator is different. To assess whether we would also observe wavelength‐dependent behavior when printing with an equal number of photons absorbed, the concentration of photoinitiator in the photoresin was adjusted to obtain four resins with the same monomer ratio (25 wt.% of PETA in PEGDA 700) and with the same absorbance (*A* = 0.4) at the wavelengths of interest. These four photoresins were subsequently used to print a 2D Printed Action Plot with the same number of photons delivered and also absorbed at all wavelengths. Pictures of the structures obtained with each photoresin are shown in Figure 3A.

Printing at 430 and 415 nm (12 and 1.5 mm photoinitiator concentration, respectively) was successful and resulted in well‐defined squares. Conversely, for 370 and 355 nm (0.015 and 0.014 mm, respectively), no curing was observed in the initial stages of the printing process, and only after a certain lag time, some curing occurred, and small irregular structures were formed. The mechanical properties of the structures printed with the same number of photons absorbed were probed by nanoindentation (Figure 3B). Conversely to what was observed for nanoindentation results obtained printing with the same number of photons delivered (Figure 2E), the structure printed at 430 nm displays the highest elastic modulus, followed by the square printed at 415 nm. Interestingly, the irregular structure obtained at 355 nm shows a higher modulus compared to the also irregular and even smaller print at 370 nm.

One possible explanation for these results is that the number of radicals initially generated is not wavelength invariant, and the radical yield that contributes to the polymerization is a function of the excitation wavelength utilized. Thus, if radical generation at 355 and 370 nm is sufficiently low, the rate of radical quenching by oxygen dominates over the rate of photopolymerization. After the oxygen is depleted, the number of radicals generated can then become sufficient to start the curing process. Meanwhile, if a higher number of radicals are formed at longer wavelengths at a higher rate, it can compete with the rate of oxygen quenching and allow for faster polymerization.

### 2.2 3D Printed Action Plot

The photocuring of one‐layer structures demonstrates that the wavelength‐dependent reactivity of a photoinitiator, measured as polymerization conversion (or photocleavage efficiency) [26] in a solution photopolymerization action plot, does not directly correlate with its reactivity in a photoresin in the context of light‐induced network formation. One of the main applications of light‐based curing is in the field of 3D printing – particularly SLA and DLP – in which multiple layers of resin are cured sequentially to form the desired 3D structure. Moving from one‐layer to multiple‐layer printing, the variable landscape becomes more complex as the curing of a layer could impact the previously formed layers, causing overcuring. Penetration depth of a light beam is a critical parameter when it comes to light‐based 3D printing [38], and varies with the excitation wavelength. However, since most printing setups do not allow to freely select printing wavelengths, laser power and layer height (z step) are carefully tailored to control overcuring of previous layers. How the specific wavelength can influence the properties of the networks and resulting materials in a 3D printing setup is explored below, where the same multi‐layer structures (two layers and ten layers) were 3D printed with our MonoLISA printer at different wavelengths, delivering the same number of photons, and characterized as the first 3D Printed Action Plot.

**FIGURE 4:**
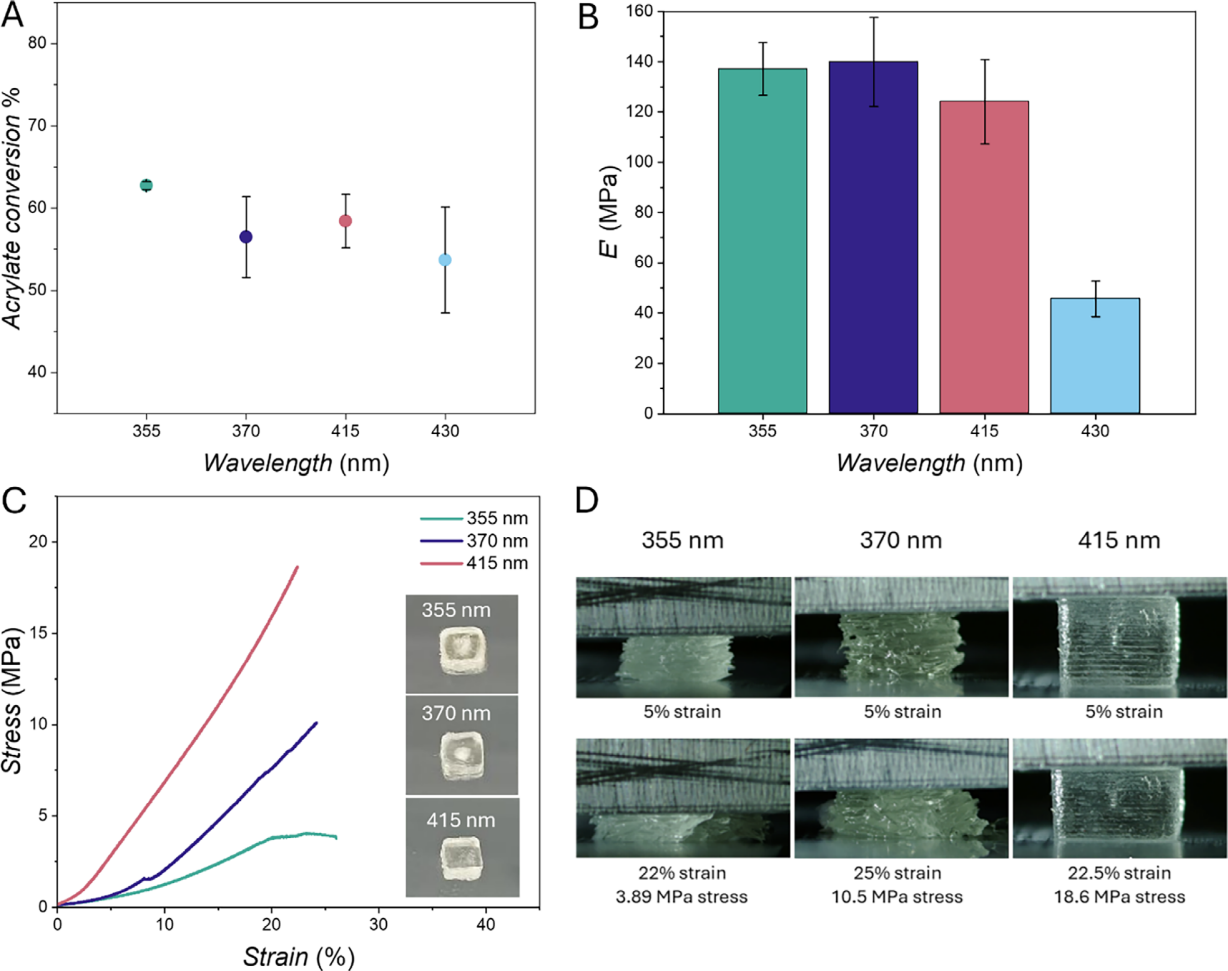
(A) Double bond conversion of two‐layer 3D Printed Action Plot delivering 4.30·10^15^ photons s^−1^ obtained via FTIR spectroscopy. Conversion of the acrylates double bond is quantified using the C═C peak = 810 cm^−1^, and C═O reference peak = 1720 cm^−1^, and averaged between measurements of top and bottom layers of the printed structures. Error bars represent standard deviations. (B) Nanoindentation of two‐layer 3D Printed Action Plot delivering 4.30·10^15^ photons s^−1^. Experiments are performed in triplicate; error bars represent standard deviations. (C) Example of compression curves of ten‐layer 3D Printed Action Plot carried out delivering 7.75·10^14^ photons s^−1^. The three cubes obtained (3 × 3 × 3 mm) are shown in (C) and printing conditions can be found in Sections S3 and S7. (D) Images extracted from real‐time deformation videos (Supporting Information, refer to the compression test videos, which form part of the Supporting Information upload).

Initially, a simpler system consisting of only two‐layer squares was 3D printed at all wavelengths to better analyze the wavelength‐dependent effects on multi‐layer overcuring via FTIR (Figure 4A). Measurements of the top and bottom layers of the squares revealed that average conversion still remains nearly identical within error across wavelengths (from 62% to 54%), consistent with the single‐layer prints (Figure 2B). Further, compared to the one‐layer nanoindentation results (Figure 2E), a stark increase in elastic modulus measured via nanoindentation for the two‐layer square cured at 415 nm is observed (Figure 4B), with elastic modulus comparable to the 355 and 370 nm prints. Notably, the elastic modulus of the structure printed at 430 nm remains approximately three times lower than the 355 nm print, similarly to the one‐layer prints (Figure 2E). 3D printing of ten‐layer cubes required optimization of the printing parameters to identify conditions that allowed printing of the same structure at all wavelengths (all printed action plots use the same G‐code for printing at all wavelengths. G‐codes of all specimens can be found in Supporting Information Section S7). Initially, the shape of the structures 3D printed with higher laser power (Figure S7) at 355 and 370 nm was distorted by light scattering effects, occurring due to the light scattering from the steel printing platform into the solution and causing unwanted curing of the photoresin around the square obtained in the center. At 415 nm, the printing is more defined, but the size of the structure is still larger than desired. Lastly, 430 nm results in a better‐defined but smaller than targeted cube. Upon decreasing laser power – and keeping the number of photons constant for all wavelengths – light intensity becomes sufficiently low that scattering from the platform back into solution is significantly reduced, avoiding curing of the area surrounding the printed structures. However, no curing occurs at 430 nm under these conditions. Compression tests of the three ten‐layer cubes (Figure 4C) obtained curing at 355, 370, and 415 nm show a completely different trend compared to the one‐layer and two‐layer prints characterization experiments. The cube printed at 415 nm appears clear, shows better shape fidelity and layer definition (Figure 4D), and does not fracture in the compression conditions used (maximum stress applicable 20 N). The 355 nm opaque cube fractures under 22% strain (3.89 MPa), and horizontal inter‐layer fractures can be identified (Figure S13). The 370 nm cube appears opaque as well, fractures around 25% strain (10.5 MPa), and vertical, intra‐layer fractures can be observed in the deformation images (Figure S13). The compressive modulus was determined as the slope of the stress‐strain curve in the initial linear elastic region, with 355 nm showing the lowest compressive modulus (18.79 ± 0.02 MPa), followed by 370 nm (28.93 ± 0.75 MPa) and 415 nm with the highest compressive modulus (75.21 ± 3.78 MPa).

This difference can be explained by the different network topology: inhomogeneous crosslinking results in highly crosslinked stiff regions of the network and low crosslinking density regions with high chain mobility that enable crystallization and contribute to the brittleness of acrylate‐based networks [45]. When the network is more homogeneous, mobility is more limited and crystallization is prevented, yielding materials that are less brittle and with improved compressive modulus.

Overall, our data demonstrate that information on in‐solution wavelength‐dependent initiation performance of a photoinitiator is not sufficient to accurately predict wavelength‐dependent network formation nor the properties of cured networks. However, a solution photopolymerization action plot study using the photoinitiator of interest is critical to obtain an accurate picture of its photoreactivity. Translating these findings to 3D printing conditions, in which the microenvironment, photoresin composition, and wavelength‐dependent parameters likely play a major role in the efficiency of network formation, demonstrates that the solution photopolymerization action plot findings cannot be directly translated into the printing realm in detail. Similarly to in‐solution action plots that are system‐specific, the influence of all the variables that constitute the microenvironment surrounding a chromophore – such as photoresin properties and composition, oxygen, scattering, penetration depth, and concentrations – is highly specific to the photoresin system. Taken together, the current knowledge on solution photopolymerization action plots and the results presented herein suggest that recording 3D Printed Action Plots (3D PAP) of a photoresin of interest is the most reliable approach to accurately investigate the reactivity and network formation of a photoresin system as a whole, as photopolymerization action plots in solution provide insights mainly on the initiation process. In addition to network formation efficiency, we find that the choice of wavelength has a significant influence on the network topology and, ultimately, material properties of the final cured structures. At the same time, it has become clear – broadly aligned with the solution photochemical action plot results – that curing the herein investigated photoresin system at longer wavelength beyond the absorption maximum of the photoinitiator results in more efficient 3D printing, with lower energetic requirements, better printing definition, and superior mechanical properties.

## 3 Conclusion

Today, photochemical action plots are considered a key methodology to access wavelength‐resolved photochemical reactivity. A plethora of studies have employed action plots to map photochemical reactivity in small‐molecule and photopolymerization solution systems. However, the influence of different chemical components of a photoresin, concentrations, presence of oxygen, light scattering, and light penetration depth are only some of the parameters that differ and have a significant influence when comparing solution‐based systems to the photocuring leading to printed structures. To date, no study has explored how the knowledge gained in solution‐based photochemical action plots translates to the realm of light‐driven 3D printing. Herein, the wavelength‐dependent photochemical crosslinking of a photoresin has been investigated, introducing the 3D Printed Action Plot (3D‐PAP) methodology. Similarly to in‐solution photochemical action plots – that are performed by delivering an identical number of photons for each specific monochromatic wavelengths followed by mapping of the chemical reactivity via chemical characterization – 3D Printed Action Plots are carried out by curing a photoresin with the same number of photons at different wavelengths followed by network and materials characterization via several analytical techniques, effectively investigating the wavelength dependent reactivity of a photoresin. The results of the Printed Action Plot of the photoresin system confirm that the overall reactivity of the resin is not fully predicted by an in‐solution photopolymerization action plot of the same photoinitiator (DMPP‐(L)‐SMe), and that the mechanical properties of the cured structures are not only related to the extent of the photoinduced crosslinking. However, there is also a clear indication that longer wavelengths on the red side of the photoresin's extinction spectrum yield superior outcomes than wavelengths that are aligned with the absorption maximum. In addition to opening an exciting new avenue for the study of wavelength‐dependent photocuring, the potential of Printed Action Plots to translate the level of precision photochemistry available for in‐solution systems to curable crosslinked networks for 3D printing paves the way to new possibilities in the realm of tailored materials. The opportunity of introducing wavelength as an additional key parameter to tune mechanical properties in 3D printed structures arises, with important implications for 3D printing in fields that would benefit from the low energy and increased penetration depth of longer wavelengths, whilst retaining high network formation efficiency.

## Conflicts of Interest

The authors declare no conflict of interest.

## Supporting information




**Supporting File**: adma73069‐sup‐0001‐SuppMat.pdf.

## Data Availability

The data that support the findings of this study are available in the supplementary material of this article.
