# Supplementary material for: Wavelength‐Dependent 3D Printing: Introducing 3D Printed Action Plots
Source: Adv Mater. 2026 Apr 16;38(27):e23664. doi: 10.1002/adma.202523664 (PMC13173393; doi:10.1002/adma.202523664)
Supplement: Supplementary file 1 — Supporting File: adma73069‐sup‐0001‐SuppMat.pdf. [file ADMA-38-e23664-s001.pdf]

Supporting Information

Wavelength-Dependent 3D Printing: Introducing 3D Printed Action Plots

Federica Sbordone, Lauren Geurds, Joshua A. Carroll, Yanan Xu, Alicia K. Finch, Filip Petko, Andrzej Świeży, Joanna Ortyl and Christopher Barner-Kowollik**

F. Sbordone, C. Barner-Kowollik
Institute of Functional Interfaces (IFG), Karlsruhe Institute of Technology (KIT), Kaiserstr. 12, 76131 Karlsruhe, Germany

F. Sbordone, L. Geurds, J. A. Carroll, A. K. Finch, C. Barner-Kowollik
School of Chemistry and Physics, Centre for Materials Science
Queensland University of Technology (QUT)
2 George Street, Brisbane, QLD 4000, Australia
E-mail: christopher.barnerkowollik@qut.edu.au

Y. Xu
Central Analytical Research Facility
Queensland University of Technology
Brisbane, Queensland 4000, Australia

F. Petko, A. Świeży, J. Ortyl
Department of Biotechnology and Physical Chemistry
Faculty of Chemical Engineering and Technology
Cracow University of Technology
Warszawska 24, Cracow 30–155, Poland
E-mail: jortyl@pk.edu.pl

Photo Hitech Ltd.

Bobrzynskiego 14

Cracow, 30-348, Poland.

Contents

Materials 3
1. In solution Action Plot Set up 5
2. Polymerization Action Plot of DMPP-(L)-SMe in solution 7
3. Monochromatic Tuneable Laser Integrated Stereolithographic Apparatus (Mono LISA) 9
4. Working curves 11
5. 2D Printed Action Plots 11
6. 3D Printed Action Plots 12
7. G-codes 14
8. Swelling tests 14
9. Scanning Electron Microscopy 14
10. UV-Vis Spectroscopy 15
11. Fourier Transform Infrared (FTIR) Spectrometry 15
12. Differential Scanning Calorimetry (DSC) 17
13. Dynamic Mechanical Analysis (DMA) 17
14. Nanoindentation 18
15. Compression tests 19
16. Estimation of Laser Induced Temperature Variation 20

Materials

All chemicals and solvents were used as received from the supplier without further purification, unless stated otherwise.

Acetone (HPLC grade, Thermo Fisher), Isopropanol (Chemsupply), water (Milli-Q, Merck), Poly(ethylene glycol) diacrylate, average Mn 700 (Sigma Aldrich), pentaerythritol triacrylate (PETA, Sigma Aldrich), Methyl methacrylate (Sigma-Aldrich. Methyl methacrylate was passed through a basic alumina column before use. Water was purified by an SP-1 MilliQ purification system.

The photoinitiator DMPP-(L)-SMe was provided by the Ortyl laboratory, used without further purification and characterized by the NMR spectra provided below. The synthetic procedure is described elsewhere.[1]

^{1}H NMR and ^{13}C NMR spectra were recorded in DMSO–d_6 using an Avance III HD 400 MHz spectrometer (Bruker). Chemical shifts (δ) are reported in parts per million (ppm) relative to tetramethylsilane (TMS) and are calibrated using the residual protonated solvent peak (δ = 2.50 ppm in ^{1}H NMR spectra and 39.52 ppm ^{13}C NMR spectra).

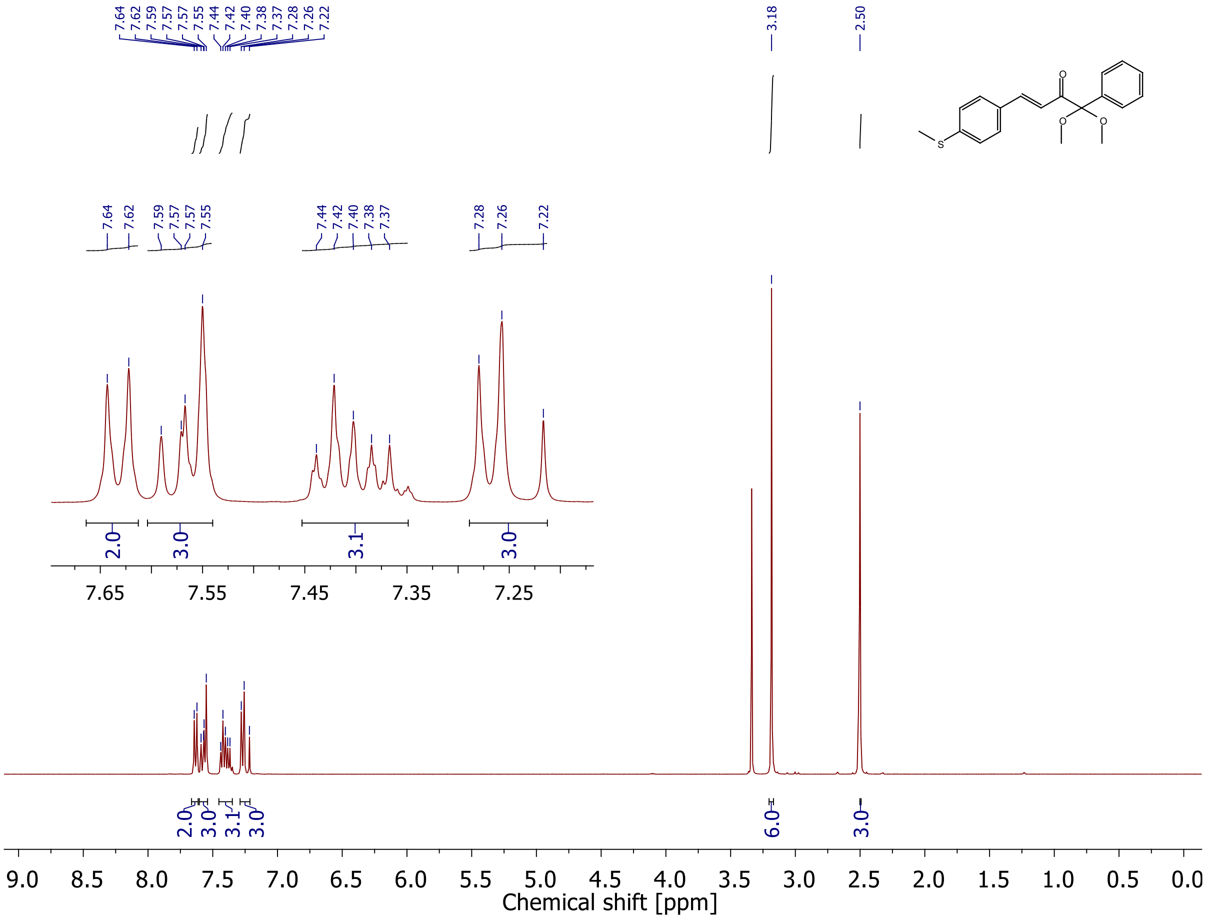

Figure S1. ^{1}H NMR spectrum of the photoinitiator DMPP-(L)-SMe as received in DMSO-d_6.

^{1}H NMR (400 MHz, DMSO) δ 7.63 (d, J = 8.4 Hz, 2H), 7.57 (m, 3H), 7.40 (m, 3H), 7.25 (m, 3H), 3.18 (s, 6H), 2.50 (s, 3H).

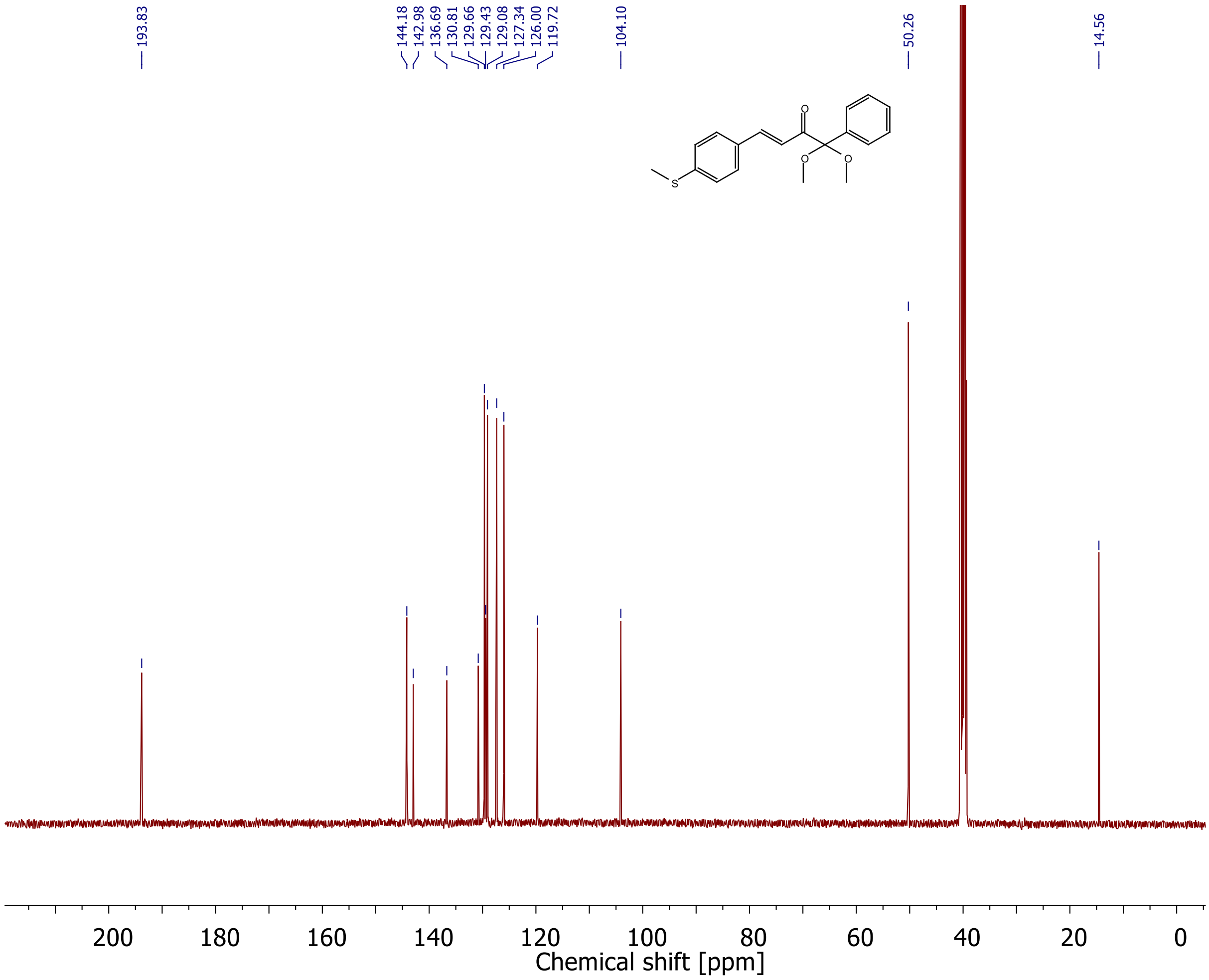

Figure S2. ^{13}C NMR spectrum of the photoinitiator DMPP-(L)-SMe as received in DMSO-d_6.

^{13}C NMR (101 MHz, DMSO) δ 193.83, 144.18, 142.98, 136.69, 130.81, 129.66, 129.43, 129.08, 127.34, 126.00, 119.72, 101.10, 50.26, 14.56

1. In solution Action Plot Set up

All laser experiments were conducted with an Opotek Opolette HE 355 LD OPO tuneable laser system with a pulse duration of 5 ns and a repetition rate of 20 Hz. For the in-solution action plot set up,[2] the emitted pulse was expanded to 7 mm diameter using a lens combination and directed upwards using a prism. The spectral linewidth (FWHM) of the beam is 4-6 cm^{-1}. The beam was redirected into the vertical cylindrical hole of a custom-made sample holder, which contains the sample vial during the experiments. The energy of the incident laser pulses was measured by a Coherent EnergyMax thermopile sensor (J-25MB-LE) placed directly above the sample holder (immediately before and after each sample was inserted into the sample holder).

Prism and sample holder are positioned such that the complete diameter of the hole of the sample holder was covered by the incident laser beam. For the irradiation experiments, all samples (0.5 mL degassed solutions used for polymerization) were prepared in 0.7 mL crimped glass vials by Lab Logistic Group GmbH (Internal diameter 5.5 mm) capped with a rubber/PTFE septum.

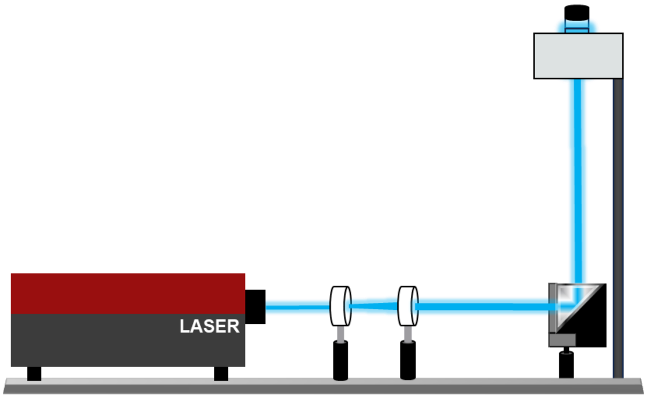

Figure S3. Schematic diagram of apparatus used for solution photopolymerization action plot.

The wavelength dependent glass transmittance, essential for quantitative measurements, is presented in Figure S4 Transmittance of the bottom of the glass vials used in the current study.

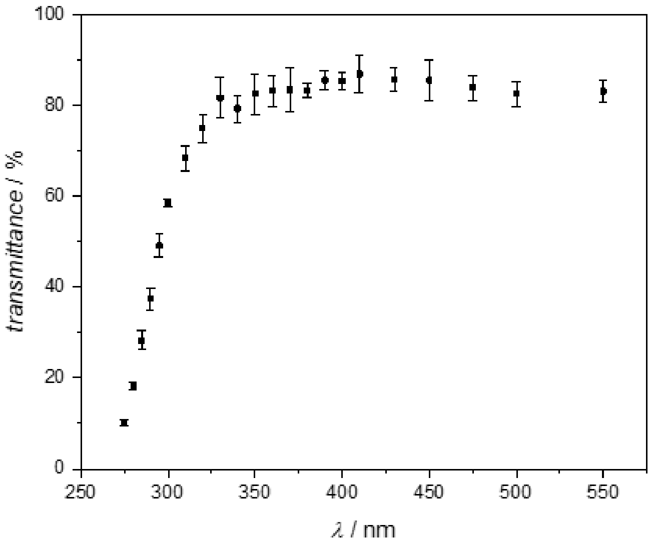

Figure S4. Transmittance of the bottom of the glass vials used in the current study.[3]

Precise photons numbers were determined from the laser pulse energy using the following relation.

$$N_p = \frac{E_{pulse}\lambda f_{rep} t}{hc[T_\lambda/100]}$$

where E_{pulse} is the measured pulse energy above the aluminium block, λ is the wavelength of the incident radiation, f_{rep} is the laser repetition rate, t is the irradiation time, h is Planck's constant, c is the speed of light and T_λ is the wavelength dependent glass transmission presented in Figure S4. Once an initial measurement is completed and the photon number is known, the required energies at other wavelengths can be found by rearranging the above equation to give E_{pulse}.

$$E_{pulse} = \frac{N_p hc\left[\frac{T_\lambda}{100}\right]}{\lambda f_{rep} t}$$

2. Polymerization Action Plot of DMPP-(L)-SMe in solution

All samples were prepared in a glovebox under nitrogen atmosphere from a stock solution with a concentration of photoinitiator $c = 5\cdot10^{-3}$ mol L^{-1} in methyl methacrylate (MMA) (sample volume 0.5 mL). Subsequently, the samples were individually placed into the sample holder and irradiated at ambient temperature. The polymerization was carried out in the tuneable laser set-up described in Section S1 with a constant photon count ($4.56\cdot10^{19}$) at each wavelength in triplicates. After irradiation, the remaining monomer was evaporated in a vacuum oven, and the monomer conversions were determined gravimetrically. For wavelengths with large energy fluctuations more replicates were performed and outliers removed.

***Wavelength* (nm)**	***E*$_0$ (μJ)**	***E*$_0$ st. dev. (%)**	***t* (min)**	***Polymer mass* (g)**	***Conversion* (%)**	***Average conversion* (%)**
325	612	1.85	60	0.022	4.78	
325	611	1.99	60	0.025	5.34	4.84±0.38
325	610	1.90	60	0.021	4.40	
340	561	2.03	60	0.023	4.91	
340	561	2.27	60	0.025	5.36	4.99±0.27
340	560	4.04	60	0.022	4.72	
355	348	3.07	90	0.027	5.74	
355	348	3.03	90	0.026	5.61	5.81±0.19
355	346	3.14	90	0.029	6.08	
370	333	2.89	90	0.035	7.40	
370	335	3.13	90	0.031	6.66	6.51±0.79
370	339	3.06	90	0.026	5.48	
385	323	3.52	90	0.030	6.40	
385	321	4.34	90	0.027	5.68	5.95±0.32
385	321	4.35	90	0.027	5.76	
400	308	5.55	90	0.029	6.19	
400	304	5.56	90	*0.040*	*8.53**	
400	304	5.55	90	0.027	5.66	5.41±0.75
400	294	5.64	90	0.018	*3.89**	
400	304	5.67	90	0.021	4.40	
415	460	4.78	60	0.036	7.59	
415	441	5.07	60	0.026	5.61	
415	445	6.16	60	0.040	8.57	7.03±1.14
415	885	4.74	30	*0.084*	*17.89**	
415	881	5.16	30	0.029	6.34	
415	875	4.64	30	*0.014*	*2.91**	
430	847	2.26	30	0.017	3.63	
430	844	2.77	30	0.017	3.70	3.79±0.18
430	847	2.41	30	0.019	4.04	
445	824	2.56	30	0.007	1.53	
445	825	1.97	30	0.007	1.46	1.46±0.05
445	826	1.78	30	0.006	1.40	

Table 1. Polymer sample overview of samples obtained by polymerizing MMA with DMPP-(L)-SMe, E_0 is the irradiation energy, irradiation time in minutes, polymer mass by subtracting residual initiator mass (0.000821 g) from the sample mass, conversion per sample relative to the initial monomer mass (0.47 g) and average conversions and standard deviations of the samples. For the 400 and 415 nm irradiation more replicates were performed and outliers excluded from calculation of average conversion. Outliers are shown in *italic* with an asterisk (*).

3. Monochromatic Tuneable Laser Integrated Stereolithographic Apparatus (Mono LISA)

All components of the 3D printing system are mounted on an optical table. As shown in Figure S5, an Opotek Opolette 355 OPO wavelength-tuneable laser producing 5 ns, 20 Hz pulses with a flattop signal profile (①) serves as the light source. The output beam reflects through UV-enhanced aluminium mirrors (③ and ④, avg. reflectance > 90%) and subsequently passes through an electronic shutter (⑤). It further passes a spherical focusing lens (⑥, f = 300 mm) mounted on a long-travel stage and directs to a 3-axis stage (⑧, Thorlabs motorized translation stage PT3-Z29 with three K-Cube DC Servo Motor Controllers) using a UV silica right angle prism (⑦, 185 nm - 2.1 μm). The power meter (②) is positioned between the laser and the first mirror to measure all powers for printing. The resin tank is placed under the X, Y and Z stage by a tank holder and the build platform with a movable bar is mounted on the X, Y and Z stage. Photographs of the printing platform including the 3-axis (X, Y and Z) stage, build platform, resin tank and tank holder as well as models of these three components, are shown in Figure S6.

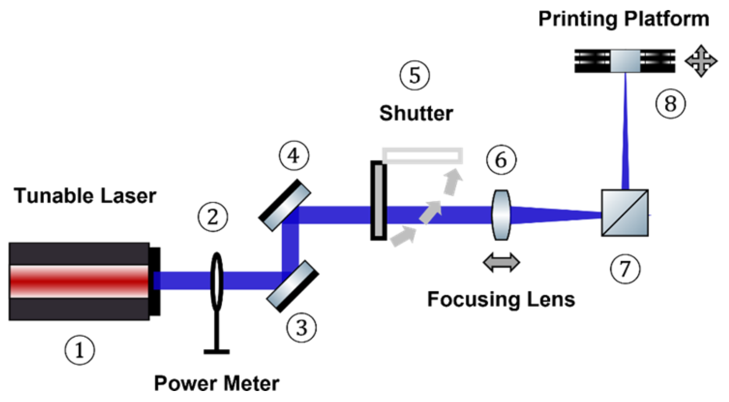

Figure S5. Schematic of the Mono LISA printer, showing the beam path and various components.[4]

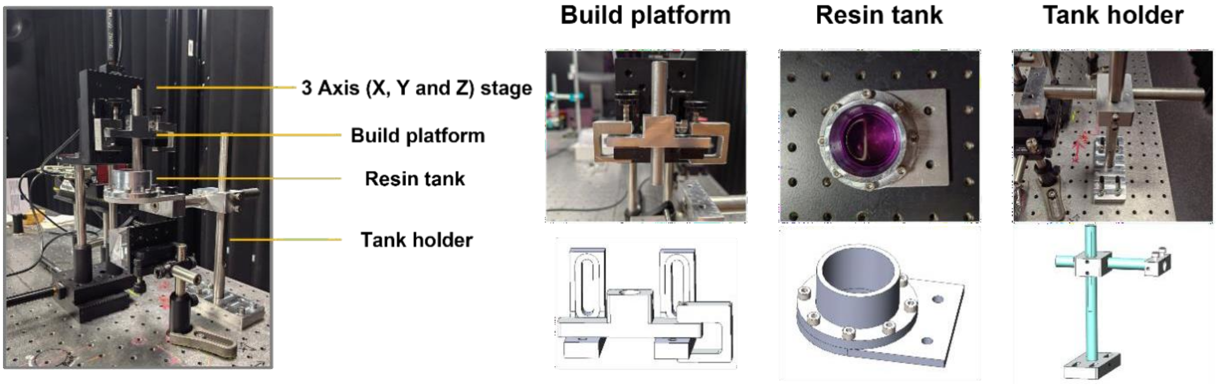

Figure S6. Photographs of the wavelength-tuneable laser printing platform and the CAD models of three components.[4]

The movement of the long-travel stage, the X, Y and Z stage as well as the shutter (on-off) is controlled by our customized software S11, programmed in LabView. Upon importing G-code files containing the coordinates and travel speed, the software processes the files to precisely control both the printing trajectory and speed. The LabView software is used to establish communication with each of the motors' controllers of the 3-axis (X, Y and Z) stage and the long-travel stage. Once communication is established, LabView can automate sending the X, Y and Z coordinates to the controllers of the 3-axis stage, allowing the three motors to move at the same time, as well as defining the velocity for each axis. Furthermore, to automate a series of commands to enable 2D and 3D printing, the LabView program is written to accept the widely used G-code file format. G-code files can be generated using commercially available software, such as UltiMaker Cura (v. 5.6.0). G-code files for the printed structures were generated using UltiMaker Cura (v. 5.6.0) and Autodesk Fusion (2025). The printing speed for the structures was 0.1 mm s^{-1} except for the rectangles in Figure 2A used for DMA analysis, printed at 0.3 mm s^{-1}. After laser exposure, all printed structures were developed by immersion in isopropanol.

For 2D Printed Action Plots the resin tank was substituted by moulds secured on glass slides and placed on the X, Y, Z stage. For all Printed Action Plots, the energy of the incident laser pulses was measured by a Coherent EnergyMax thermopile sensor (J-25MB-LE) placed immediately after the UV silica right angle prism ⑦.

4. Working curves

Working curves were obtained printing 2×2 mm squares at different incident radiant exposure values (E_0). E_0 was varied by increasing laser power and keeping printing time and speed constant. Thickness of the printed structures was measured with a MITUTOYO 103-137 micrometre screw gauge.

Wavelength **(nm)**	***Equation***	***E_c (mJ cm^{-2})***	***D_p (mm)***
355	y=0.153ln(x)-0.891	344	0.153±0.06
370	y=0.354ln(x)-2.191	485	0.354±0.08
415	y=1.389ln(x)-6.650	119	1.389±0.30
430	y=3.824ln(x)-20.540	214	3.824±0.42

Table 2. Jacobs working curves obtained for at each wavelength 0.5 wt% of DMPP-(L)-SMe and 25 wt% PETA in PEGDA 700.

5. 2D Printed Action Plots

2D Printed Action Plots were recorded using the set-up described in Section 3. 800 µL of photoresist were poured into the mould secured on the glass slide. Wavelength dependent transmittance (T) of the glass slides, essential for quantitative measurements, has been determined as above (Section S1). For the preliminary Printed Action Plots (lines, Figure 1C) a range of energy and number of photons delivered were explored, presented in Table 3. All following 2D Printed Action Plots were carried out delivering $1.42 \cdot 10^{16}$ photons per second, corresponding to the third row of lines in Figure 1C and Table 3. All structures were printed with a printing speed of 0.1 mm s^{-1}, except for the rectangles in Figure 2A used for DMA analysis, printed at 0.3 mm s^{-1}.

Wavelength (nm)	*Line* (top to bottom)	E_0 (μJ)	E_0 st. dev. (%)	*T* (%)	*N* of photons s^{-1}
355	1	595	3.03	86.2	$1.84 \cdot 10^{16}$
	2	530	3.01		$1.63 \cdot 10^{16}$
	3	462	2.99		$1.42 \cdot 10^{16}$
	4	400	3.27		$1.23 \cdot 10^{16}$
	5	330	2.97		$1.02 \cdot 10^{16}$
370	1	555	3.15	89.3	$1.84 \cdot 10^{16}$
	2	491	4.08		$1.63 \cdot 10^{16}$
	3	428	3.20		$1.42 \cdot 10^{16}$
	4	370	3.31		$1.23 \cdot 10^{16}$
	5	305	3.14		$1.02 \cdot 10^{16}$
415	1	480	6.01	91.8	$1.84 \cdot 10^{16}$
	2	426	5.62		$1.63 \cdot 10^{16}$
	3	371	5.81		$1.42 \cdot 10^{16}$
	4	321	5.65		$1.23 \cdot 10^{16}$
	5	265	5.54		$1.02 \cdot 10^{16}$
430	1	471	2.78	90.2	$1.84 \cdot 10^{16}$
	2	418	2.98		$1.63 \cdot 10^{16}$
	3	364	2.80		$1.42 \cdot 10^{16}$
	4	315	2.83		$1.23 \cdot 10^{16}$
	5	260	2.81		$1.02 \cdot 10^{16}$

Table 3. Overview of laser parameters used for 2D Printed Action Plots. Structures were obtained by curing of a photoresist composed of 0.5 wt% of DMPP-(L)-SMe and 25 wt% PETA in PEGDA 700, E_0 is the irradiation energy. Each 2D Printed Action Plot conducted using the same G-Code for all wavelengths.

6. 3D Printed Action Plots

3D Printed Action Plots were produced using the set up described in Section S3. 10 mL of photoresist composed of 0.5 wt% of DMPP-(L)-SMe and 25 wt% PETA in PEGDA 700 were poured into the vat. Wavelength-dependent transmittance of the fluorinated ethylene propylene (FEP) film that constitutes the bottom of the vat, essential for quantitative measurements, has been determined as above (Section S1). The number of photons delivered to each print before (set 1, Figure S7) and after (set 2, Figure 4C) optimization of printing conditions is reported in Table 4.

Wavelength (nm)	Set	E_0 (µJ)	E_0 st. dev. (%)	*T* (%)	*N* of photons s^{-1}
355	1	157	3.82	76.4	$4.30 \cdot 10^{15}$
	2	28	3.53		$7.75 \cdot 10^{14}$
370	1	145	3.45	79.2	$4.30 \cdot 10^{15}$
	2	26	3.42		$7.75 \cdot 10^{14}$
415	1	122	5.41	84.3	$4.30 \cdot 10^{15}$
	2	22	5.53		$7.75 \cdot 10^{14}$
430	1	115	2.90	86.3	$4.30 \cdot 10^{15}$
	2	20.7	2.78		$7.75 \cdot 10^{14}$

Table 4. Overview of laser parameters used for 3D Printed Action Plots. Structures were obtained by curing of a photoresist composed of 0.5 wt% of DMPP-(L)-SMe and 25 wt% PETA in PEGDA 700, E_0 is the irradiation energy. Each 3D Printed Action Plot was carried out using the same G-Code for all wavelengths. Printing at 430 nm with $7.75 \cdot 10^{14}$ photons s^{-1} resulted in no curing.

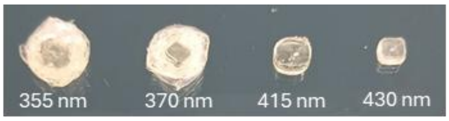

Figure S7. 3D Action Plot printed with $4.30 \cdot 10^{15}$ photons s^{-1}.

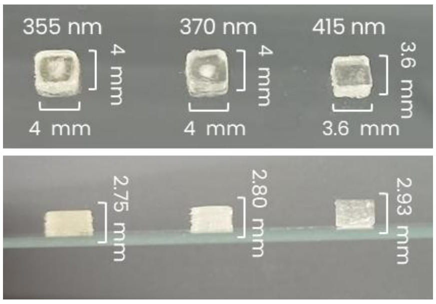

Figure S8. 3D Action Plot printed with $7.75 \cdot 10^{14}$ photons s^{-1}. Dimensions in G-code represent a 3×3×3 mm cube.

7. G-codes

Printed action plots were recorded using the same G-Code file for every wavelength. For 3D printed structures the z step was also kept constant (z = 0.2 mm). Images illustrating the trajectory of the 3-axis (X, Y and Z) stage for the squares, rectangles and cubes, generated from the G-code files to obtain the corresponding structures are shown below.

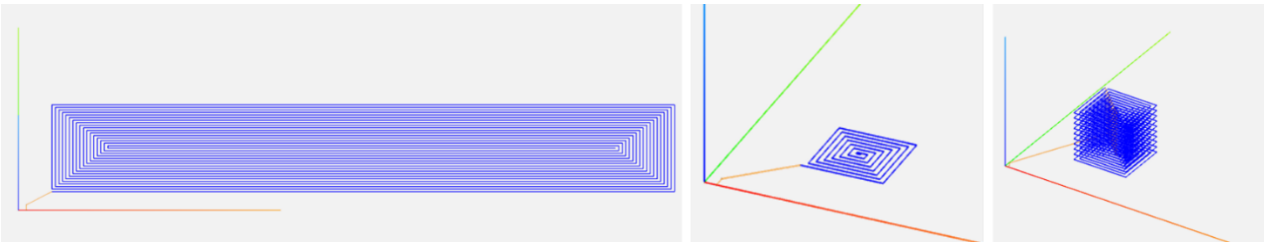

Figure S9. Trajectory of the 3-axis (X, Y and Z) of the G-code files used. The blue lines represent the trajectory when the shutter is on (i.e., resin exposure to the laser). The orange lines show the trajectory when the shutter is off (i.e., resin is not exposed to the laser).

8. Swelling tests

Swelling tests were performed on 2×2 mm printed squares. After development in isopropanol, the samples were soaked in acetone overnight, dried in a vacuum oven and weighted to obtain dry weight (w_d) and swelled in MilliQ water for 48 hours. The samples were subsequently removed from water and weighted to obtain swollen weight (w_s). Experiments were performed in triplicates; error bars represent standard deviation. Swelling ratio (S_r) was calculated as follows:

$$S_r = \frac{w_s - w_d}{w_d}$$

9. Scanning Electron Microscopy

SEM images were captured using a Tescan MIRA3 at 10 kV equipped with a secondary electron detector. Each sample was coated with a 2 nm layer of platinum.

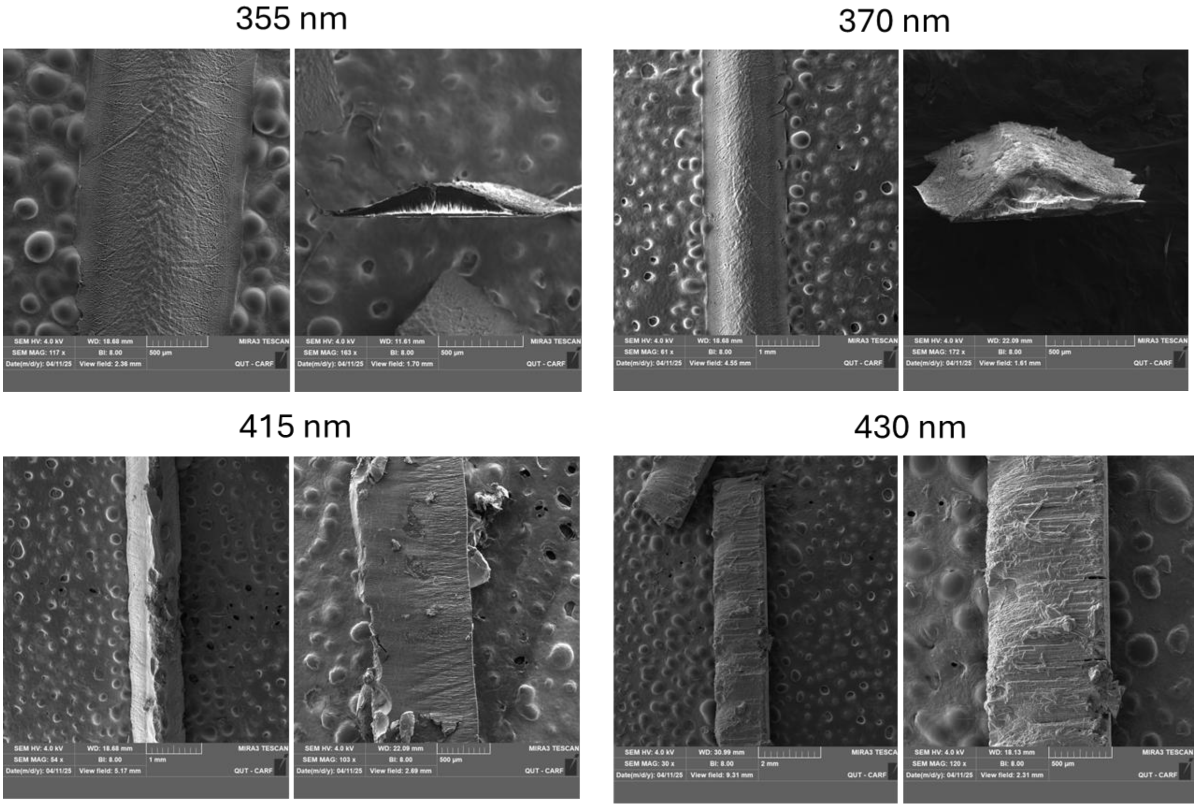

Figure S10. SEM images of lines printed with $1.99{\cdot}10^{18}$ photons

10. UV-Vis Spectroscopy

UV-VIS spectra were recorded on a Shimadzu UV-2700 spectrophotometer equipped with a CPS-100 electronic temperature control cell positioner. Samples were measured in Hellma Analytics quartz high precision cells with a path length of 1 cm at ambient temperature

11. Fourier Transform Infrared (FTIR) Spectrometry

All IR measurements were performed on a *Bruker* Alpha-P ATR-IR from 500-4000 cm^{-1} at ambient temperature. Conversion of the acrylate double bond was calculated on the area of the C=C peak at 810 cm^{-1} and normalized to the area of the C=O peak at 1720 cm^{-1} as below:

$$DC\% = \left[1 - \frac{A_{(810)} \;/\; A_{(1720)}}{A_{0(810)} \;/\; A_{0(1720)}}\right] \times 100$$

where A_0 is the area of the peaks of the uncured photoresist. Measurements were performed in triplicates; error bars represent standard deviation. Representative spectra of the 2D action plot are reported below in Figure S11.

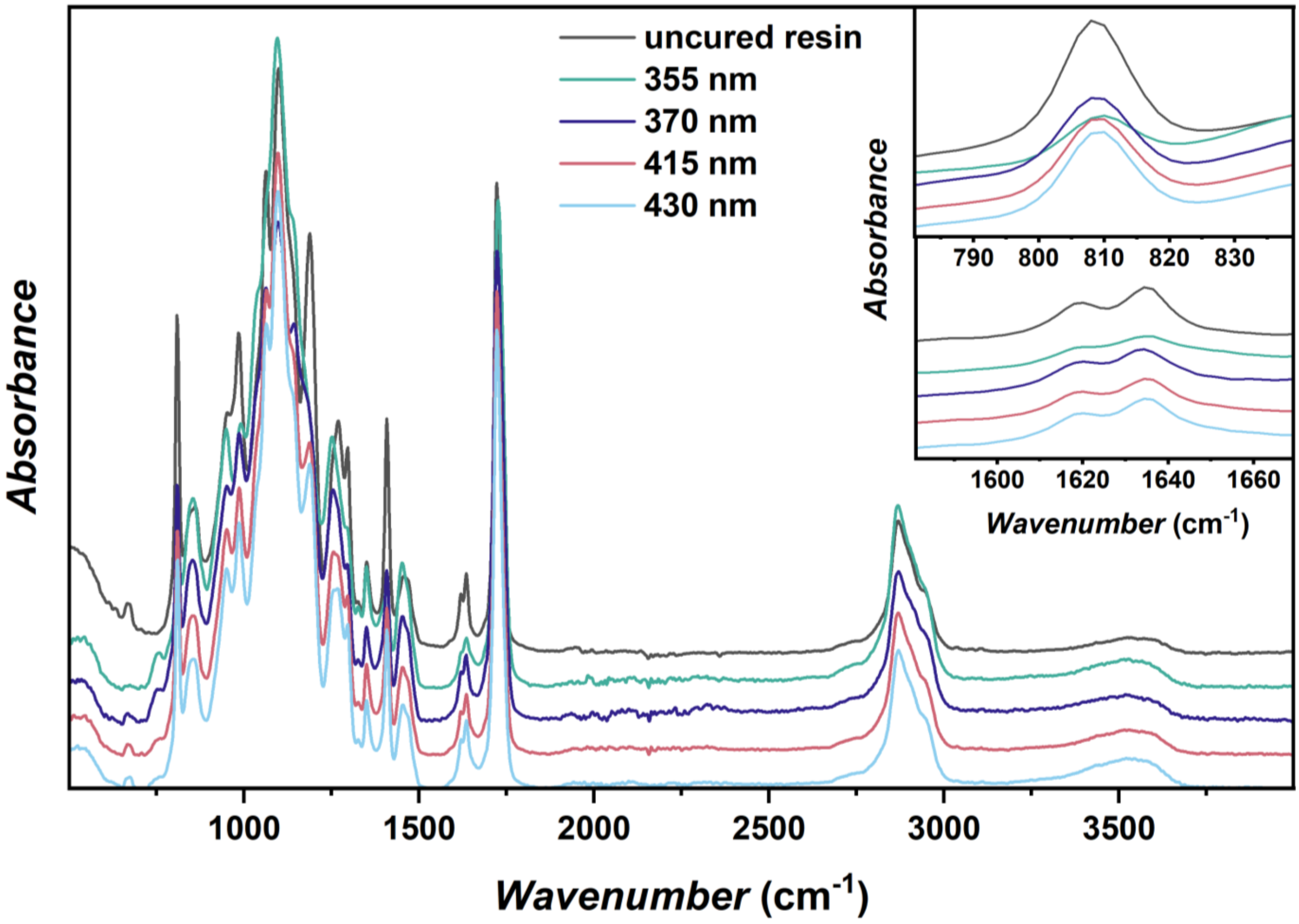

Figure S11. Overlay of the FTIR spectra of uncured resin and structures 2D printed at 355, 370. 415 and 430 nm delivering $1.42 \cdot 10^{16}$ photons s^{-1}. The spectra have been stacked and thus the y-axis labels have been removed. All spectra are baselined. The double bond peak at 810 cm^{-1} (top inset) was used for quantification of the monomer consumption. The double bond peak at 1630 cm^{-1} is also shown in the bottom inset.

12. Differential Scanning Calorimetry (DSC)

DSC measurements were performed to determine the thermal transitions of the 3D printed samples. All measurements were conducted using a *Netzsch Phoenix DSC* (204 F1) equipped with an automated sample changer and an intracooler with a temperature range of -90 to 500 *°C*. Samples (typically 5–10 mg) were accurately weighed and sealed in standard aluminium pans and pierced before measurement; identical empty pans were used as references.

Each sample was subjected to a standard heating–cooling–reheating protocol. The samples were first heated from -90 to 250 C at a rate of 10 K min^{-1} to erase previous thermal history, followed by cooling back to -90 C at the same rate. A second heating cycle was subsequently performed under identical conditions. To determine glass transition temperatures (T_g), melting transitions, crystallisation behaviour, and/or residual cure enthalpy, the two heating processes were evaluated.

Thermal transitions were extracted from both heating scan unless stated otherwise. Data processing and baseline corrections were performed using Netzsch Proteus Analysis software.

13. Dynamic Mechanical Analysis (DMA)

DMA was performed to evaluate the viscoelastic properties of the 2D printed samples. Measurements were conducted using a TA Instruments DMA 850 equipped with a tensile geometry clamp. Rectangular specimens with dimensions of approximately e.g., 20×5 mm were 2D printed. Thickness of all rectangles was measured with a caliper, to account for variations resulting from different wavelength penetration depths. Samples were subjected to a temperature sweep from -90 to 250 °C at a heating rate of 1 °C min^{-1}, using a constant oscillation frequency of 1 Hz and a strain amplitude within the linear viscoelastic regime (typically 0.05%). Preliminary strain sweep experiments were performed to confirm linearity.

The storage modulus (*E*'), loss modulus (*E*''), and loss factor (tan δ) were recorded as functions of temperature. The glass transition temperature (T_g) was determined from the peak of tan δ or from the onset of the storage modulus as shown in the figure below.

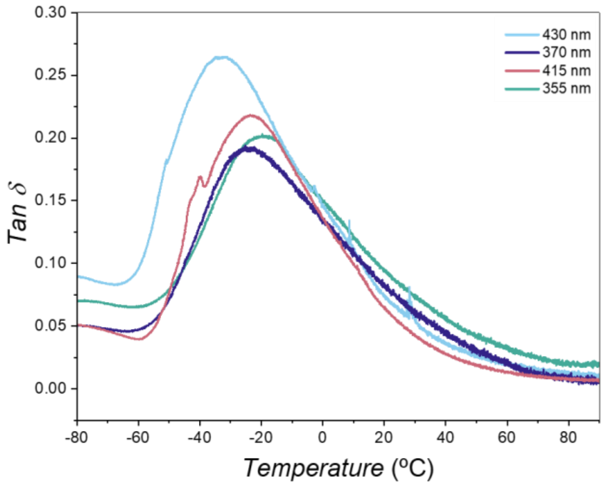

Figure S12. Tan δ plot of 2D rectangles shown in Figure 2A, printed with $1.42\cdot10^{16}$ photons s^{-1}. The printing conditions are described in Section 5.

14. Nanoindentation

Nanoindentation experiments were performed using a Hysitron TI 950 Triboindenter equipped with a cono-spherical diamond tip of 5 µm radius at room temperature. Tests were conducted under displacement control to a maximum indentation depth of 2000 nm, with loading and unloading rates of 400 nm s^{-1} and 800 nm s^{-1}, respectively, and a dwell time of 15 s at maximum depth. The load–displacement data were continuously recorded during testing, and the reduced modulus was calculated using the Oliver–Pharr method:[5]

$$E_r = \frac{\sqrt{\pi}}{2}\frac{S}{\sqrt{A}}$$

where *S* is the measured stiffness, *A* is the contact area. E_r is a combined modulus of the sample and the indenter, which is determined by:

$$\frac{1}{E_r} = \frac{(1-v^2)}{E} + \frac{(1-{v_i}^2)}{E_i}$$

where, E and v are the elastic modulus and Poisson's ratio of the sample, E_i and v_i are the elastic modulus and Poisson's ratio for the indenter. For a standard diamond indenter probe, E_i is 1140 GPa and v_i is 0.07. Poisson's ratio of the sample was assumed to be 0.35.

15. Compression tests

Uniaxial compression tests were conducted using an MTS Tytron 250 system equipped with a 250 N load cell. To minimize end effects caused by friction, a thin layer of silicone oil was applied to the surfaces of grips. Testing was performed under displacement control at a crosshead speed of 1 mm min^{-1}. Axial force and axial deformation were recorded, and the engineering stress σ and engineering strain ε were calculated as:

$$\sigma = \frac{F}{A_0}, \varepsilon = \frac{\Delta L}{L_0}$$

where, F is the applied force, A_0 is the initial cross-sectional area, ΔL is the axial deformation, and L_0 is the initial sample height. Real-time deformation was captured using a high-resolution camera, and the comparative results are shown in Figure S13. The compressive modulus was determined as the slope of the stress-strain curve in the initial linear elastic region. Measurements were performed in duplicates.

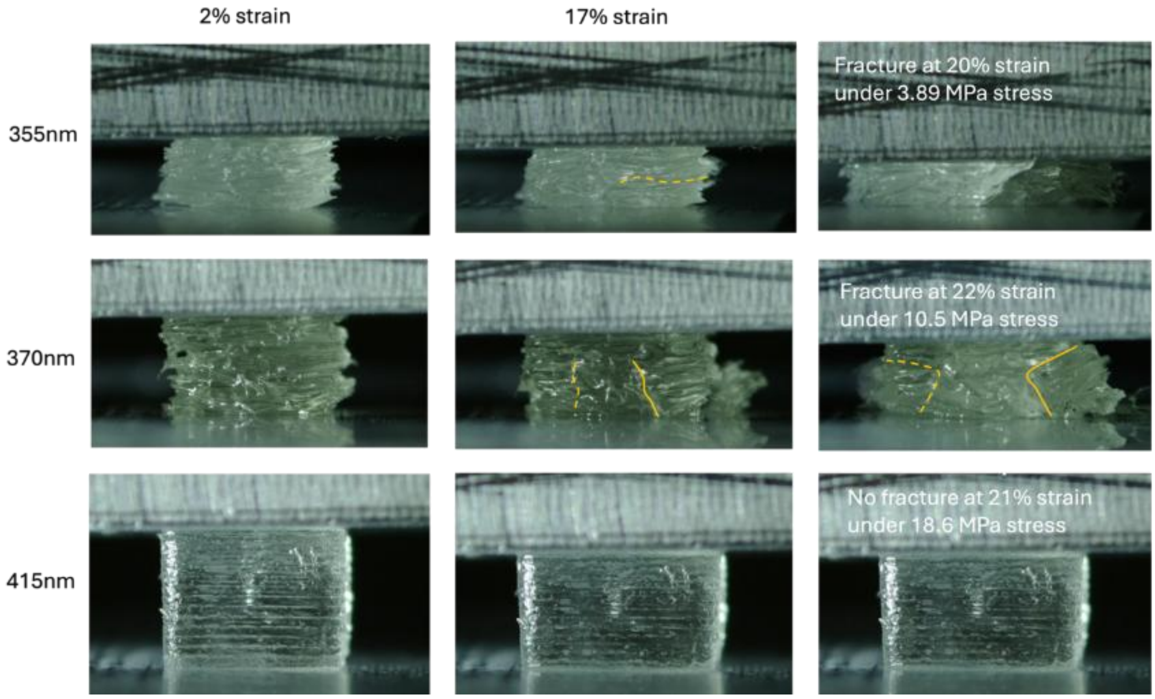

Figure S13. Deformation of samples cured with 7.75·10^{14} photons s^{-1} at different strain levels. Yellow lines highlight the fractures detected.

16. Estimation of Laser Induced Temperature Variation

Calculations on a PEG600 based system have been performed to elucidate whether laser irradiation can induce relevant temperature changes over time. PEG600 was chosen as the closest derivative with reported specific heat capacity and thermal diffusivity to the PEGDA700 used in this study.

Assuming the entire pulse energy at the highest photon flux of 462 µJ/pulse at 355 nm was entirely absorbed by the sample, based on the reported specific heat capacity[6] of PEG600 of $c_{PEG} = 2.5\,J/(g\cdot K)$ and a sample solution of 10 mL, the expected temperature increase across the solution is expected to be

$$\Delta T = \frac{E_{pulse}}{m_{PEG600}\ c_{PEG600}} = \frac{462\times 10^{-6}J}{\left(1.12\frac{g}{mL}\times 10\ mL\times 2.5\,J/(g\cdot K)\right)} = 0.000016\,K.$$

This calculated temperature increase is cumulative across the entire solution per pulse, assuming the worst-case scenario that all the absorbed energy is converted to heat. It is therefore also worthwhile to consider the local heating under the same assumption and the thermal diffusion times.

At the concentrations used in our current study (12 mM), we expect there to be an average separation L between the chromophores of close to 5.15 nm. Taking the reported thermal conductivity of PEG600[6] of κ_{PEG} = 0.2 W/m K, the average temperature increase around each molecule which absorbs a 355 nm photon is expected to be

$$\Delta T = \frac{E_{photon}}{mc_{PEG600}}$$

$$= \frac{E_{photon}}{\rho_{PEG600}L^3c_{PEG600}} = \frac{5.6\times 10^{-19}J}{1120\ Kg/m^3\times 1.37\times 10^{-25}\ m^3\times 2500J/Kg\cdot K} = 1.46\,K.$$

The thermal diffusion time can be calculated by $\tau = L^2/\alpha$ where $\alpha = \kappa_{PEG600}/[\rho_{PEG600} c_{PEG600}]$ is the thermal diffusivity. Using the previously mentioned solvent parameters, the thermal diffusion time is

$$\tau = \frac{L^2}{\alpha} = \frac{L^2}{\left[{}^{\kappa_{PEg600}}/_{\rho_{PEG600} c_{PEG600}}\right]} = 3.7 \times 10^{-10} s.$$

Given the laser has a low repetition rate of 20 Hz, leaving 50 ms between successive pulses, we can reasonably assume that thermal effects are negligible in the current study.

References

[1] A. Świeży, F. Petko, D. Krok-Janiszewska, P. Szymaszek, M. Galek, J. Ortyl, "New Efficient D-π-A and A-π-A Structured Type I Radical Photoinitiators for Additive Manufacturing Nanomaterials Preparation" *Adv Mater Technol* 2025, *10,* 2402139.

[2] I. M. Irshadeen, S. L. Walden, M. Wegener, V. X. Truong, H. Frisch, J. P. Blinco, C. Barner-Kowollik, "Action Plots in Action: In-Depth Insights into Photochemical Reactivity" *J Am Chem Soc* 2021, *143,* 21113–21126.

[3] D. Kanchana, J. A. Carroll, N. Giacoletto, D. Gigmes, J. Kim, A. N. Unterreiner, K. Mundsinger, B. T. Tuten, C. Barner-Kowollik, "Wavelength-Resolved Oxime Ester Photoinitiator Decay in Radical Polymerization" *Macromolecules* 2024, *57,* 9779–9787.

[4] X. Wu, K. Ehrmann, C. T. Gan, B. Leuschel, F. Pashley-Johnson, C. Barner-Kowollik, "Two Material Properties from One Wavelength-Orthogonal Photoresin Enabled by a Monochromatic Laser Integrated Stereolithographic Apparatus (Mono LISA)" *Advanced Materials* 2025, *37,* 2419639.

[5] W. C. Oliver, G. M. Pharr, "An improved technique for determining hardness and elastic modulus using load and displacement sensing indentation experiments" *J Mater Res* 1992, *7,* 1564–1583.

[6] Minea, A. A. State of the Art in PEG-Based Heat Transfer Fluids and Their Suspensions with Nanoparticles. *Nanomaterials* **2021**, *11* (1), 86.
